# Association between exclusive or dual use of combustible cigarettes and heated tobacco products and depressive symptoms

**DOI:** 10.1371/journal.pone.0314558

**Published:** 2025-01-03

**Authors:** Bo Gyeong Lee, Haein Lee, Namhee Kim

**Affiliations:** 1 College of Nursing, Daegu Catholic University, Daegu, Republic of Korea; 2 Department of Nursing, Hanseo University, Seosan-Si, Republic of Korea; Kurdistan University of Medical Sciences, ISLAMIC REPUBLIC OF IRAN

## Abstract

**Purpose:**

Despite the advent of heated tobacco products (HTPs), their relationship to mental health remains unclear. This study aimed to determine associations between the use of combustible cigarettes (CCs) and HTPs with depressive symptoms.

**Methods:**

This descriptive-analytical cross-sectional study was conducted in March 2023. Using the 8th Korea National Health and Nutrition Examination Survey, 5,349 adults aged 19 years or older were classified into four groups: non-users, CC-only users, HTP-only users, and dual users. Relationships between exclusive or dual use of CCs and HTPs and depressive symptoms were analyzed using item scores and total scores of the Patient Health Questionnaire-9 (PHQ-9). To examine associations between exclusive or dual use of CCs and HTPs and depressive symptoms, a multinomial regression analysis was performed using the PHQ-9 total score.

**Results:**

HTP-only users had the highest proportion of those with anhedonia and depressed mood. CC-only users had the highest proportion of individuals with trouble sleeping, while dual users had a higher proportion of those with fatigue and appetite problems. After adjusting for general characteristics, compared to non-users, CC-only users were more likely to have mild and moderate to severe depressive symptoms. HTP-only users and dual users were also more likely to have moderate to severe depressive symptoms.

**Conclusions:**

All smokers have a higher risk of depression than non-smokers. Health care providers should closely monitor depressive symptoms, especially in HTP users and dual users of tobacco products.

## Introduction

The association of cigarette smoking with depressive symptoms has been well documented [1–3]. Many previous studies have shown that nicotine tolerance can cause smokers to experience anxiety and depressive symptoms and disrupt dopamine levels, leading to depression or major depressive disorder [2–4]. Despite these significant relationships between smoking and mental health, recent evolution of tobacco products has led smokers to try various tobacco products [5]. Therefore, the association between use of various tobacco products and mental health should be carefully investigated from a public health perspective.

Heated Tobacco Products (HTPs) are heat-not-burn cigarettes in which tobacco leaves are heated with a battery to create an aerosol, which the smoker inhales [6]. Although the aerosol produced by HTPs, like combustible cigarettes (CCs), contains highly addictive nicotine and flavoring substances [6], the market of HTPs has grown explosively due to aggressive marketing by manufacturers and the misconception that they are less harmful than CCs [7, 8]. In fact, 5.1% of adults in Korea and 10.9% of adults in Japan use HTPs [7, 9]. Total sales of HTPs in 2016 were US$2.1 billion. They were expected to reach US$17.9 billion in 2021 [10]. However, since HTPs have recently been introduced into the tobacco market, there is controversy over the harmfulness of HTPs. According to some previous studies, certain hazardous substances such as nitrosamines, acetaldehyde, and formaldehyde are likely to be contained in higher concentrations in HTPs than in CCs [11, 12]. Therefore, HTPs are becoming a major public health concern.

Many studies have been conducted to examine the awareness of HTPs [11, 13] and physical problems of HTPs, including respiratory and cardiovascular risks [14]. However, studies on relationships between HTPs and psychological problems are insufficient. Only a few studies have reported an association between adolescent use of HTPs and suicide-related risk behaviors [15, 16]. Given that many smokers choose HTPs to reduce toxicity of CCs or to reduce health risks as a means to quit smoking [7, 8], the psychological effects of HTPs need further investigation.

Meanwhile, as tobacco products become more diverse, smokers not only use one product, but also use multiple products simultaneously [5]. This poly-tobacco use causes many side effects, including increased risks of smoking-related diseases and nicotine dependence [17, 18]. According to a previous study, compared to exclusive users of CCs, dual users of both CCs and electronic cigarettes (ECs) show poorer psychological quality of life and social skills [18]. This result suggests that dual or poly-tobacco use might have a more negative impact on mental health than single use. Research results on HTPs from previous studies are controversial. In addition, there are very few studies comparing depressive symptoms using HTPs only and dual use of tobacco products including HTPs with traditional cigarettes. Therefore, we analyzed the association between exclusive or dual use of CCs and HTPs and depressive symptoms. For this purpose, data from the Korea National Health and Nutrition Examination Survey (KNHANES Ⅷ-2) that ensured representativeness of the sample were used.

## Materials and methods

### Study design and participants

In this cross-sectional study, data from the 8th (2020) KNHANES Ⅷ-2 were analyzed to determine the association between exclusive or dual use of CCs and HTPs and depressive symptoms [19]. KNHANES is a nationwide health and nutrition survey conducted annually to assess public health behavior, the prevalence of chronic diseases, and food and nutritional intake. KNHANES employs a two-stage stratified cluster sampling method, with national surveys and households as sampling units, targeting individuals aged 1 year or older living in South Korea [19]. In the present study, data from 5,349 adults aged 19 or older who responded to both smoking-related and mental health questionnaires were used. The KNHANES Ⅷ-2 data set was acquired on March, 2023. KNHANES data are released to the general public for research purposes and do not contain personally identifiable data. As this study involved the analysis of secondary data, obtaining written consent from participants was not required. This study obtained approval for exemption from review by Daegu Catholic University Institutional Review Board. All research procedures complied with the Declaration of Helsinki.

### Instruments

#### Depressive symptoms

In this study, we assessed depressive symptoms using the Patient Health Questionnaire-9 (PHQ-9) [20]. Although PHQ-9 is the gold standard for depression screening, the optimal cutoff for the total score to balance specificity and sensitivity remains unclear [21, 22]. Therefore, depressive symptoms were examined using not only using the total score of the PHQ-9 but also at the item level based on previous studies [21]. In this way, the prevalence of anhedonia, depressed mood, and suicidal ideation, which are key items for diagnosing depressive symptoms—key items for diagnosing depressive symptoms—according to exclusive or dual use of CCs and HTPs. In addition, according to a recent study on PHQ-9, a bi-factor model consisting of six items of cognitive/affective factor and three items of somatic factor was found to be appropriate for non-clinical populations [21]. Therefore, PHQ-9 was divided into cognitive/affective and somatic factors in the present study. The PHQ-9 consists of nine questions asking respondents to what extent they have experienced depressive symptoms over the past two weeks [20]. Response choices were: never at all = 0, several days = 1, more than half the days = 2, and nearly every day = 3. The algorithm for diagnosing depression using PHQ-9 is as follows [20, 22]: a) major depression disorder is diagnosed when five or more of the nine items (including anhedonia or depressed mood) are present for more than half the days; b) other depression can be diagnosed if symptoms appear for more than half the days in 2 to 4 with symptom of anhedonia or depressed mood; c) regardless of symptom duration, depression is diagnosed when suicidal ideation occurs; and d) as a way to determine the level of depression severity, a total score of 4 or less is classified as minimal, 5 to 9 points as mild, 10 to 14 points as moderate, 15 to 19 points as moderately severe, and 20 to 27 points as severe. The PHQ-9 has shown good reliability and validity in many previous studies, including the Korean version [20, 23, 24]. The Cronbach’s alpha coefficients was 0.81 in the present study. A previous study has shown that an item level cutoff point of 2 for five items is appropriate for depression screening in non-clinical populations [22]. That study also reported that the item level cutoff point for suicidal ideation, at 1 was the most consistent with scoring recommendations [22]. Therefore, we used an item level cutoff point of 2 for all items except suicidal ideation.

#### Exclusive or dual use of CCs and HTP

Participants were classified into four groups based on their use of CCs or HTPs in the past month. Participants who reported never having used CCs and HTPs in their lifetime or had used them in the past but were not currently using them were classified as non-users. For questions regarding use of CCs or HTPs, participants who reported daily or occasional use of one but had never or did not currently use the other were classified as CC-only users or HTP-only users, respectively. Finally, participants who currently used both CCs and HTPs daily or occasionally were classified as dual users.

#### General characteristics

General characteristics included gender, age, education level, occupation, household income, physical activity, body mass index (BMI), drinking days per month, perceived stress level, and sleep duration. Occupations were classified into office workers, service and sales workers, manual labor workers, and no occupation. Household income was classified into low, middle (lower middle and upper middle), and high using income quartile data. Regular physical activity was defined as more than 150 minutes of moderate-intensity physical activity or more than 75 minutes of high-intensity physical activity per week [19]. According to obesity treatment guidelines recommended by the Korea Society for the Study of Obesity, BMI was classified as 22.9 kg/m^2^ or less (underweight, normal), 23.0–24.9 kg/m^2^ (pre-obesity), and 25.0 kg/m^2^ or more (obesity) [25]. The number of drinking days and perceived stress level were reclassified based on the number of drinking days per month and a dichotomous variable, respectively. Perceived stress level was categorized into those who felt ’a lot’ of stress in their daily lives and those who did not. According to the National Sleep Foundation, 7–9 hours of sleep per day is appropriate for normal adults while, and less than 6 hours is not recommended [26]. Therefore, sleep duration was classified as ’sufficient’ and ’insufficient’ based on 6 hours of sleep per day.

#### Data analysis

Analyses for complex survey samples were conducted using IBM SPSS Statistics version 25.0 (IBM Corp., Armonk, NY, USA). To generalize analysis results of this study to the target population, strata, clusters, and weights were considered. Weighted percentage and cross-tabulation were used to compare differences in general characteristics and depressive symptoms by PHQ-9 items according to exclusive or dual use of CCs and HTPs. In addition, multinomial logistic regression analysis was performed using total score of the PHQ-9 to examine the association between exclusive or dual use of CCs and HTPs and depression severity. In Model Ⅰ, the relative risk was calculated without adjusting for any variables. In Models II and III, variables shown to have significant differences between groups among general characteristics were adjusted. Numerous previous studies have shown that perceived stress level and sleep duration are associated with depressive symptoms [27, 28]. Thus, these variables were further adjusted in Model III.

## Results

### General characteristics of participants according to exclusive or dual use of CCs and HTPs

Of the total of 5,349 participants, 81.8% were non-users, 15.2% were CC-only users, 1.2% were HTP-only users, and 1.8% were dual users. Those aged 51 years or older had the highest proportion at 37.6% in CC-only users. In dual users, the age group of 30 years or less had the highest proportion at 40.7% (χ^2^ = 205.07, *p* < .001). The proportion of those with a college or higher education was 62.8% in HTP-only users and 53.0% in dual users (χ^2^ = 40.69, *p* < .001). Manual labor workers had the highest proportion at 34.1% in CC-only users. Office workers had the highest proportions in HTP-only users at 48.2% (χ^2^ = 171.28, *p* < .001). In terms of perceived stress, it was 43.9% in HTP-only users and 46.8% in dual users (χ^2^ = 53.85, *p* < .001). Regarding insufficient sleep duration, 21.7% of CC-only users and 25.4% of dual users (χ^2^ = 14.50, *p* = .032) (Table 1).

**Table 1:** General characteristics according to exclusive or dual use of combustible cigarette and heated tobacco products. (N = 5,349).

Variables	Categories	Non-users (n = 4,377)		CC-only users (n = 812)		HTP-only users (n = 66)		Dual users (n = 94)		χ^2^	*p*
		**Unweighted frequency (unweighted %)**									
**Sex**	Male	1,613	(40.9)	669	(84.8)	54	(82.7)	81	(89.4)	699.48	< .001
	Female	2,764	(59.1)	143	(15.2)	12	(17.3)	13	(10.6)		
**Age (yr)** ^**a**^	≤30	637	(19.1)	139	(19.9)	14	(21.2)	37	(40.7)	205.07	< .001
	31–40	592	(16.3)	130	(20.4)	25	(46.2)	25	(30.2)		
	41–50	687	(17.9)	158	(22.1)	18	(23.7)	20	(19.3)		
	≥51	2,461	(46.8)	385	(37.6)	9	(8.9)	12	(9.7)		
**Education level**	≤High school	2,680	(56.8)	550	(64.6)	26	(37.2)	43	(47.0)	40.69	< .001
	≥College	1,697	(43.2)	262	(35.4)	40	(62.8)	51	(53.0)		
**Occupation**^**a**^ **(n = 5,336)**	Office worker	1,070	(28.2)	177	(24.4)	32	(48.2)	39	(38.7)	171.28	< .001
	Service and sales personnel	585	(13.8)	119	(15.6)	12	(19.3)	18	(21.0)		
	Manual labor	864	(18.6)	282	(34.1)	9	(14.4)	16	(16.7)		
	No occupation	1,849	(39.5)	233	(25.8)	13	(18.0)	18	(23.6)		
**Household income**^**a**^ **(n = 5,335)**	Low	762	(13.9)	138	(14.4)	4	(5.2)	6	(6.4)	22.25	.036
	Middle	2,257	(51.3)	434	(53.7)	29	(46.8)	46	(47.5)		
	High	1,346	(34.8)	238	(32.0)	33	(47.9)	42	(46.1)		
**Physical activity**	Regular^b^	1,841	(43.9)	319	(41.8)	28	(42.2)	49	(57.5)	11.65	.043
	Irregular	2,536	(56.1)	493	(58.2)	38	(57.8)	45	(42.5)		
**BMI (kg/m** ^**2**^ **) (n = 5,282)**	≤22.9	1,735	(40.0)	282	(32.9)	18	(25.3)	29	(27.1)	36.13	.002
	23–24.9	963	(22.5)	204	(25.8)	15	(24.3)	19	(19.7)		
	≥25.0	1,621	(37.5)	318	(41.3)	33	(50.4)	45	(53.2)		
**Drinking days per month**	No drinking	1,485	(29.4)	98	(9.7)	8	(14.6)	6	(4.7)	485.43	< .001
	≤1 day	1,340	(32.2)	164	(20.4)	10	(15.2)	23	(23.8)		
	2–4 days	870	(21.6)	211	(27.3)	15	(16.1)	35	(34.8)		
	≥5 days	682	(16.8)	339	(42.6)	33	(54.1)	30	(36.7)		
**Perceived stress level**	Low	3,255	(73.1)	540	(65.2)	38	(56.1)	48	(53.2)	53.85	< .001
	High	1,122	(26.9)	272	(34.8)	28	(43.9)	46	(46.8)		
**Sleep duration** ^**c**^ **, weekday**	Sufficient	3,529	(82.4)	635	(78.3)	57	(87.3)	69	(74.6)	14.50	.032
	Insufficient	848	(17.6)	177	(21.7)	9	(12.7)	25	(25.4)		

*Note*. CC = combustible cigarettes; HTP = heated tobacco products; BMI = body mass index

^a^Sums may not total 100 due to rounding.

^b^Regular physical activity = 150 minutes/week more of moderate-intensity or 75 minutes/week more of vigorous-intensity physical activity.

^c^Sleep duration ≥6 hours = sufficient; <6 hours = insufficient

### Differences in depressive symptoms according to exclusive or dual use of CCs and HTPs

The distribution of depressive symptoms with a score of 2 or more for PHQ-9 items according to CCs and HTPs use was as follows. Among cognitive/affective factor, anhedonia was reported at a high proportion of 11.5% in HTP-only users and 9.9% in dual users (χ^2^ = 7.13, *p* < .001). Depressed mood was found in 3.5% of CC-only users, 7.5% of HTP-only users, and 3.6% of dual users, with HTP- only users having the highest proportion (χ^2^ = 5.72, *p* = .001). Feeling bad about oneself was found in 4.3% of CC-only users, 4.9% of HTP-only users, and 7.1% of dual users (χ^2^ = 68.23, *p* < .001). Suicidal ideation with a cutoff score of 1 or higher was reported by 7.4% of CC-only users and 9.9% of dual users, with dual users having a higher proportion (χ^2^ = 21.08, *p* = .007) (Table 2).

**Table 2:** Comparison of the prevalence of depressive symptoms by patient health questionnaire Items following exclusive or dual use of combustible cigarette and heated tobacco products. (N = 5,349).

Factors	Items	Item/ total scores	Non-users (n = 4,377)		CC-only users (n = 812)		HTP-only users (n = 66)		Dual users (n = 94)		χ^2^	*p*
			Unweighted frequency (weighted %)									
**Cognitive/ affective**	Anhedonia	<2	4,236	(96.4)	756	(93.6)	60	(88.5)	86	(90.1)	7.13	< .001
		≥2	141	(3.6)	56	(6.4)	6	(11.5)	8	(9.9)		
	Depressed mood	<2	4,297	(98.3)	782	(96.5)	62	(92.5)	90	(96.4)	5.72	.001
		≥2	80	(1.7)	30	(3.5)	4	(7.5)	4	(3.6)		
	Feeling bad about oneself	<2	4.322	(98.9)	773	(95.7)	63	(95.1)	89	(92.9)	68.23	< .001
		≥2	55	(1.1)	39	(4.3)	3	(4.9)	5	(7.1)		
	Trouble concentrating	<2	4,325	(98.8)	792	(97.7)	64	(95.8)	92	(96.7)	14.26	.074
		≥2	52	(1.2)	30	(2.3)	2	(4.2)	2	(3.3)		
	Motor activity	<2	4,349	(99.4)	798	(98.6)	66	(100.0)	93	(98.4)	9.04	.139
		≥2	28	(0.6)	14	(1.4)	0	(0.0)	1	(1.6)		
	Suicidal ideation	<1	4,167	(95.6)	745	(92.6)	64	(95.3)	85	(90.1)	21.08	.007
		≥1	210	(4.4)	67	(7.4)	2	(4.7)	9	(9.9)		
**Somatic**	Trouble sleeping	<2	4,021	(92.7)	719	(88.8)	60	(91.2)	85	(89.4)	16.86	.021
		≥2	356	(7.3)	93	(11.2)	5	(8.8)	9	(10.6)		
	Fatigue	<2	4,056	(92.7)	731	(89.7)	61	(88.9)	82	(84.1)	21.96	.015
		≥2	321	(7.3)	81	(10.3)	5	(11.1)	12	(15.9)		
	Appetite	<2	4,258	(97.5)	768	(95.0)	64	(95.6)	87	(90.2)	35.66	< .001
		≥2	119	(2.5)	44	(5.0)	2	(4.4)	7	(9.8)		
**Total**	Depressive symptoms^a^	0–4	3,639	(83.1)	618	(75.4)	49	(71.6)	69	(72.1)	68.93	< .001
		5–9	547	(12.7)	124	(16.4)	11	(16.7)	14	(13.2)		
		10–27	191	(4.2)	70	(8.2)	6	(11.7)	11	(14.7)		

*Note*. CC = combustible cigarettes; HTP = heated tobacco products

^a^Using patient health questionnaire-9 total score, 0–4 = minimal; 5–9 = mild; 10–27 = moderate to severe depressive symptoms.

Regarding somatic factors, the proportion of those reporting depressive symptoms with a cutoff score of 2 or higher was as follows. Trouble sleeping was found in 11.2% of CC-only users and 10.6% of dual users, higher than 7.3% of non-users and 8.8% of HTP-only users (χ^2^ = 16.86, *p =* .021). Fatigue was found in 7.3% of non-users, 10.3% of CC-only users, 11.1% of HTP-only users, and 15.9% of dual users (χ^2^ = 21.96, *p =* .015). Dual users had the highest proportion in 9.8% of those who had appetite symptoms, followed by CC-only users at 5.0%, HTP-only users at 4.4%, and non-users at 2.5% (χ^2^ = 35.66, *p* < .001). Dual users had the highest proportion at 14.7% in those having moderate to severe depressive symptoms, followed by 11.7% in HTP-only users, 8.2% in CC-only users, and 4.2% in non-users (Table 2).

### Association between exclusive or dual use of CCs and HTPs and depressive symptoms

Compared to non-users, CC-only users were more likely to have mild (crude relative risk [cRR] = 1.42, 95% confidence interval [CI] = 1.13–1.79) and moderate to severe (cRR = 2.14, 95% CI = 1.57–2.92) depressive symptoms. HTP-only users (cRR = 3.21, 95% 95% CI = 1.37–7.56) and dual users (cRR = 3.99, 95% CI = 1.90–8.36) were more likely to have moderate to severe depressive symptoms compared to non-users. As a result of adjusting for general characteristics in Model Ⅱ, compared to non-users, CC-only users were more likely to have mild (adjusted relative risk [aRR] = 1.73, 95% CI = 1.31–2.30) and moderate to severe (aRR = 3.04, 95% CI = 2.01–4.60) depressive symptoms. HTP-only users (aRR = 5.41, 95% CI = 2.20–13.46) and dual users (aRR = 6.15, 95% CI = 2.70–13.99) were more likely to have moderate to severe depressive symptoms. Results of Model III, which additionally adjusted for perceived stress level and sleep duration, revealed that CC-only users were more likely to have mild (aRR = 1.55, 95% CI = 1.15–2.07) and moderate to severe (aRR = 2.29, 95% CI = 1.52–3.45) depressive symptoms compared to non-users. HTP-only users (aRR = 4.39, 95% CI = 1.54–12.54) and dual users (aRR = 4.02, 95% CI = 1.75–9.24) were more likely to have moderate to severe depressive symptoms compared to non-users (Table 3).

**Table 3:** Association between exclusive or dual use of combustible cigarette and heated tobacco products and depressive symptoms. (N = 5,349).

		Depressive symptoms^a^											
		Model Ⅰ				Model Ⅱ^b^				Model Ⅲ^b^^c^			
		Mild		Moderate to severe		Mild		Moderate to severe		Mild		Moderate to severe	
		Crude RR (95% CI)	*p*	Crude RR (95% CI)	*p*	Adjusted RR (95% CI)	*p*	Adjusted RR (95% CI)	*p*	Adjusted RR (95% CI)	*p*	Adjusted RR (95% CI)	*p*
**Sex**	Male					0.54 (0.42, 0.70)	< .001	0.44 (0.31, 0.62)	< .001	0.58 (0.45, 0.70)	< .001	0.53 (0.37, 0.73)	< .001
	Female					Ref.		Ref.		Ref.		Ref.	
**Age (yr)**	≤30					1.60 (1.20,2.09)	.001	3.29 (2.13, 5.07)	< .001	1.48 (1.11,1.98)	.008	2.93 (1.85, 4.65)	< .001
	31–40					2.24 (1.62, 3.10)	< .001	2.65 (1.65, 4.26)	< .001	1.92 (1.34, 2.76)	< .001	1.97 (1.17, 3.31)	.011
	41–50					1.03 (0.78, 1.37)	.833	1.91 (1.21, 3.02)	.006	0.96 (0.71, 1.29)	.764	1.53 (0.95, 2.48)	.081
	≥51					Ref.		Ref.		Ref.		Ref.	
**Education level**	≤High school					1.36 (1.08, 1.73)	.011	1.36 (0.94, 1.95)	.103	1.40 (1.09, 1.80)	.008	1.43 (0.99, 2.06)	.058
	≥College					Ref.		Ref.		Ref.		Ref.	
**Occupation** **(n = 5,336)**	Office worker					0.94 (0.72, 1.21)	.612	0.42 (0.27, 0.67)	< .001	0.78 (0.60, 1.02)	.072	0.33 (0.20, 0.54)	< .001
	Service and sales personnel					0.68 (0.79, 0.94)	.021	0.54 (0.34, 0.85)	.009	0.60 (0.42, 0.85)	.004	0.45 (0.27, 0.76)	.003
	Manual labor					0.63 (0.46, 0.87)	.005	0.42 (0.27, 0.67)	< .001	0.57 (0.41, 0.79)	.001	0.35 (0.21, 0.59)	< .001
	No occupation					Ref.		Ref.		Ref.		Ref.	
**Household income** **(n = 5,335)**	Low					1.65 (1.17, 2.33)	.005	3.46 (2.16, 5.57)	< .001	1.63 (1.15, 2.31)	.007	3.50 (0.21, 0.59)	< .001
	Middle					1.05 (0.81, 1.35)	.733	1.71 (1.16, 2.50)	.006	1.04 (0.80, 1.34)	.783	1.75 (1.16, 2.63)	.007
	High					Ref.		Ref.		Ref.		Ref.	
**Physical activity**	Regular^d^					0.95 (0.76, 1.18)	.637	1.05 (0.77, 1.43)	.771	0.94 (0.75, 1.18)	.604	1.05 (0.78, 1.42)	.756
	Irregular					Ref.		Ref.		Ref.		Ref.	
**BMI (kg/m** ^**2**^ **)** **(n = 5,282)**	≤22.9					1.03 (0.82, 1.30)	.808	0.74 (0.53, 1.03)	.771	1.09 (0.86, 1.39)	.483	0.84 (0.60, 1.17)	.291
	23–24.9					0.85 (0.65, 1.09)	.197	0.69 (0.45, 1.07)	.071	0.90 (0.69, 1.18)	.442	0.81 (0.51, 1.27)	.352
	≥25.0					Ref.		Ref.		Ref.		Ref.	
**Drinking days per month**	No drinking					0.88 (0.64, 1.22)	.453	1.08 (0.69, 1.70)	.725	0.92 (0.66, 1.28)	.612	1.18 (0.75, 1.86)	.470
	≤1 day					0.97 (0.69, 1.36)	.864	0.92 (0.59, 1.43)	.710	0.96 (0.68, 1.35)	.794	0.89 (0.57, 1.39)	.602
	2–4 days					1.02 (0.76, 1.38)	.893	0.94 (0.60, 1.47)	.789	1.09 (0.81, 1.47)	.576	1.02 (0.65, 1.59)	.941
	≥5 days					Ref.		Ref.		Ref.		Ref.	
**Perceived stress level**	Low									0.22 (0.18, 0.26)	< .001	0.56 (0.04, 0.08)	< .001
	High									Ref.		Ref.	
**Sleep duration** ^**e**^ **, weekday**	Sufficient									0.80 (0.65, 0.99)	.041	0.58 (0.40, 0.85)	.005
	Insufficient									Ref.		Ref.	
**Non-users** **(n = 4,377)**		Ref.		Ref.		Ref.		Ref.		Ref.		Ref.	
**CC-only users (n = 812)**		1.42 (1.13, 1.79)	.003	2.14 (1.57, 2.92)	< .001	1.73 (1.31, 2.30)	< .001	3.04 (2.01, 4.60)	< .001	1.55 (1.15, 2.07)	.004	2.29 (1.52, 3.45)	< .001
**HTP-only users (n = 66)**		1.53 (0.70, 3.37)	.286	3.21 (1.37, 7.56)	.008	1.90 (0.81, 4.44)	.137	5.41 (2.20, 13.46)	< .001	1.78 (0.76, 4.18)	.186	4.39 (1.54, 12.54)	.006
**Dual users** **(n = 94)**		1.20 (0.62, 2.32)	.579	3.99 (1.90, 8.36)	< .001	1.48 (0.73, 3.00)	.273	6.15 (2.70, 13.99)	< .001	1.18 (0.56, 2.50)	.662	4.02 (1.75, 9.24)	.001

*Note*. CC = combustible cigarettes; HTP = heated tobacco products; RR = relative risk; CI = confidence interval

^a^The reference = minimal depression symptoms (patient health questionnaire-9 total score 0–4)

^b^Adjusted for gender, age, education level, occupation, house income, physical activity, BMI, and drinking days per month.

^c^Additionally adjusted for perceived stress level, and weekday sleep duration.

^d^Regular physical activity = 150 minutes/week more of moderate-intensity or 75 minutes/week more of vigorous-intensity physical activity.

^e^Sleep duration ≥6 hours = sufficient; <6 hours = insufficient

## Discussion

In this study, we analyzed the association between exclusive or dual use of tobacco products, including recently released HTPs, and depressive symptoms using large-scale national survey data. For 7 items of the PHQ-9, the level of depressive symptoms was significantly higher in smoking groups than in non-users. In particular, anhedonia and depressed mood were approximately twice as prevalent in smoking groups than in non-users. Additionally, around 10% of HTP-only users and dual users were found to experience anhedonia more than half the days, showing a higher proportion than in non-users and even CC-only users. These two items are crucial for diagnosing major depressive disorder among the nine items of the PHQ-9 [20, 23]. These results may indicate the severity of depressive symptoms in smokers, especially in HTP-only users and dual users. Furthermore, even when total score of the PHQ-9 was evaluated, the proportion of those with mild or higher depressive symptoms ranged from 24.6% to 28.4% across smoking groups. In particular, more than 11% of HTP-only users and dual users were found to have moderate to severe depressive symptoms. These results consistently support more severe levels of depressive symptoms in HTPs uses and dual users.

Additional analysis showed that compared to non-users, all smoking groups were more likely to have moderate to severe depressive symptoms. After adjusting for general characteristics, HTP-only users were most likely to show depressive symptoms, followed by dual users. In a similar study, Han [29] has found that the prevalence of depression among adults currently using HTPs is more than twice that of adults who do not use HTPs at all. Another study has shown that HTPs users are 1.44 to 2.30 times more likely to have depressive symptoms, suicidal ideation, depressive disorder, or a diagnosis of depression compared to non-smokers and 1.79 to 3.68 times more likely than CCs users [30]. Some users of non-traditional tobacco products with relatively low nicotine content, such as HTPs, might use multiples at a time until they reach the desired nicotine level [31]. Although this study analyzed secondary data which made it impossible to determine smoking frequency, we noted that HTPs use was strongly associated with depression.

It is well known that smoking is significantly associated with poor mental health, including stress, depression, suicidal thoughts, and poor sleep quality [18, 28, 32]. If tolerance to nicotine has developed, smokers become more dependent on nicotine because anxiety, nervousness, and depression appear as nicotine levels decrease [4]. HTPs are new tobacco products developed recently, and there is still uncertainty about the relationship between HTPs and nicotine levels [10]. However, one study has shown that HTPs do not differ from CCs in pharmacokinetics or nicotine delivery [33]. Also, Lau et al. [34] have reported that daily use of HTPs is highly likely to cause nicotine dependence. These results suggest that frequent use of tobacco products, including HTPs, is associated with higher levels of nicotine in the body. Dual users of tobacco products are more vulnerable as they are known to use cigarettes more frequently than single users. HTPs have fewer environmental restrictions than CCs because they have no odor or ash [5]. For this reason, CCs users can selectively use HTPs in limited situations [5]. Ultimately, smokers are likely to use HTPs more frequently due to the misconception that HTPs are safe and their relatively liberal regulation [7, 8]. These results may become a more serious public health problem in that many smokers perceive HTPs as less harmful and less addictive than CCs and use them as a means to quit smoking [35].

Meanwhile, this study showed that the three smoking groups had higher levels of perceived stress than non-users. In particular, the perceived stress level was very high in HTP-only users and dual users. In a similar study, Seo et al. [29] have found that lifetime HTPs users have a higher level of perceived stress than non-tobacco users and lifetime CC-only users. They have also reported that dual users who used both CCs and HTPs showed higher stress levels than CC-only users [29]. In addition, in this study, dual users had the highest proportion of insufficient sleep duration. Similarly, Lee & Lee [27] have also reported that exclusive users of CCs or ECs have poor sleep satisfaction and insufficient sleep duration compared to non-smokers and that these sleep indicators are worse in dual users. Considering these findings, dual use of tobacco products might have a significant relationship not only on depressive symptoms but also on several aspects of mental health.

This study has several limitations. First, because this study was cross-sectional, there were limitations in deriving causal relationships between variables. Second, as this study was a secondary data analysis study, participant’s actual amount of smoking could not be included in the analysis. Therefore, future research is proposed to determine the amount of smoking according to the type of tobacco product use and analyze the association between these factors, nicotine concentration, and mental health. Third, as this study divided groups according to current CCs and HTPs use, retrospective data on tobacco product use (e.g., lifetime use but current temporary cessation) were not considered. Lastly, in this study, the sample size for some PHQ-9 items for each group was biased. Therefore, future studies should evaluate the validity of the cutoff point of the PHQ-9 to measure depression in smokers. This study had the advantage of generalizing research results because it analyzed representative National Health and Nutrition Examination Survey data through a large-scale national survey. Additionally, it was significant in that it was one of the few studies that confirmed psychological risks associated with HTPs.

## Conclusions

HTP-only users and dual users were more likely to have moderate to severe depressive symptoms. This study shows that the use of HTPs, like other tobacco products, has a significant association with depressive symptoms. Therefore, the level of depressive symptoms in smokers who use HTPs or dual users of tobacco products should be investigated from a public health perspective. Also, healthcare providers are required to revise awareness of HTPs by educating the public about harmful effects of HTPs. In addition, policy supplementation is needed to more strictly control the use of HTPs from a public health perspective.
